# Evaluation of YouTube Video Content About Developmental Dysplasia of the Hip

**DOI:** 10.7759/cureus.9557

**Published:** 2020-08-04

**Authors:** Ahmet Oztermeli, Nazim Karahan

**Affiliations:** 1 Orthopedics and Traumatology, Gebze Fatih Government Hospital, Izmit, TUR; 2 Orthopedics and Traumatology, Corlu State Hospital, Tekirdag, TUR

**Keywords:** developmental dysplasia of the hip, information, internet, parent education, quality, hip, video, youtube

## Abstract

Objective

The purpose of this study is to investigate the quality and reliability of YouTube videos regarding developmental dysplasia of the hip (DDH).

Background

YouTube is one of the most popular websites used as a source of information, but the variety in authorship and lack of a peer-review process are problems.

Methods

The search string “developmental dysplasia of the hip” was inputted to the YouTube search engine, and the first 52 videos returned as a response were assessed. The Video Power Index (VPI) (like ratio*view ratio/100) was used to assess the popularity of the videos. Global Quality Score (GQS) and DDH scores (DDHS) were used to evaluate the quality and educational quality of the videos, and the Journal of the American Medical Association Score (JAMAS) was used to evaluate the accuracy of the source of information.

Results

According to our research, the mean duration time of the videos was 526 seconds (SD: 813), and the average view count of the videos was 34,644. The mean time since upload was 1,907 days (SD: 1,137). On average, the videos received 10.9 comments, 210.3 likes, and 6.8 dislikes. The mean like ratio and VPI were 92.9 (SD: 19.57) and 25.8 (SD: 53.43), respectively. The mean JAMAS, GQS, and DDHS of all videos evaluated were 1.37 (SD: 0.7), 2.46 (SD: 1.09), and 4.63 (SD: 5.00), respectively. The DDHS and GQS were positively correlated (p: 0.001; r: 65.8%). The GQS and the DDHS were higher in the academic group than in the commercial group (p: 0.01 and p: 0.037, respectively).

Conclusions

The videos regarding DDH on YouTube generally had poor quality. As a result, to maintain an optimal parent-physician or patient-physician relationship, we suggest that international health societies make their own educational videos for parents, patients, and fellow physicians.

Level of evidence

Level 3.

## Introduction

The term “developmental dysplasia of the hip” (DDH) represents a wide spectrum of hip disorders such as hip instability, subluxation, dislocation, and dysplasia [1,2]. DDH is seen in 1%-1.5% of newborns, is more common in girls (5 per 1,000 in boys and 13 per 1,000 in girls), and can cause complications such as osteoarthritis and limb length discrepancy [3]. Given that DDH is one of the most frequent disorders in newborns and can cause serious complications, many concerned parents want to learn more about the disease.

In recent years, parents have been able to easily access a large source of information regarding the diseases that affect their children, thanks to the Internet. YouTube is one of the most popular websites used as a source of information [4]. According to the information obtained from YouTube, the site is visited by more than one billion Internet users every month, and 300 hours of video content is uploaded every minute [4]. This amount of material makes YouTube a very large online visual library.

Although easy access to information through YouTube can seem to make life easier, the variety in authorship and the lack of a peer-review process on YouTube are big problems. This situation could mean that parents access not only some adequate information but also some inadequate information regarding their children’s condition, which could possibly affect their decisions regarding their children’s health [5,6]. This situation means that it is essential to evaluate the quality and the reliability of YouTube videos.

Uploading videos to YouTube is easy and free of charge, and therefore video quality and reliability vary. Choosing an appropriate video to watch and from which to receive information is challenging for parents. YouTube video quality on various medical topics has been investigated in the literature, but the quality of YouTube videos regarding DDH has not been investigated thus far [7-9]. The purpose of this study is to investigate the quality and reliability of YouTube videos, specifically regarding DDH.

## Materials and methods

The search string “developmental dysplasia of the hip” was inputted to the YouTube search engine, and the first 52 videos returned as a response were assessed. Videos in a non-English language were excluded. The number of views, number of comments, number of likes, number of dislikes, the running time of the videos, and the time since the videos were uploaded were recorded.

The like ratio (like*100/[like + dislike]) and the view ratio (number of views/days) were calculated, and the Video Power Index (VPI) (like ratio*view ratio/100) was used to assess the popularity of the videos [7].

**Figure 1:**
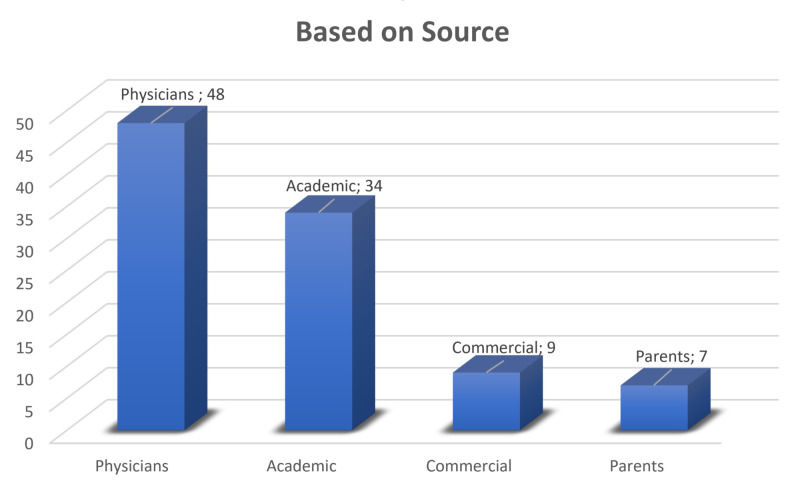
Distribution of the groups based on source

**Figure 2:**
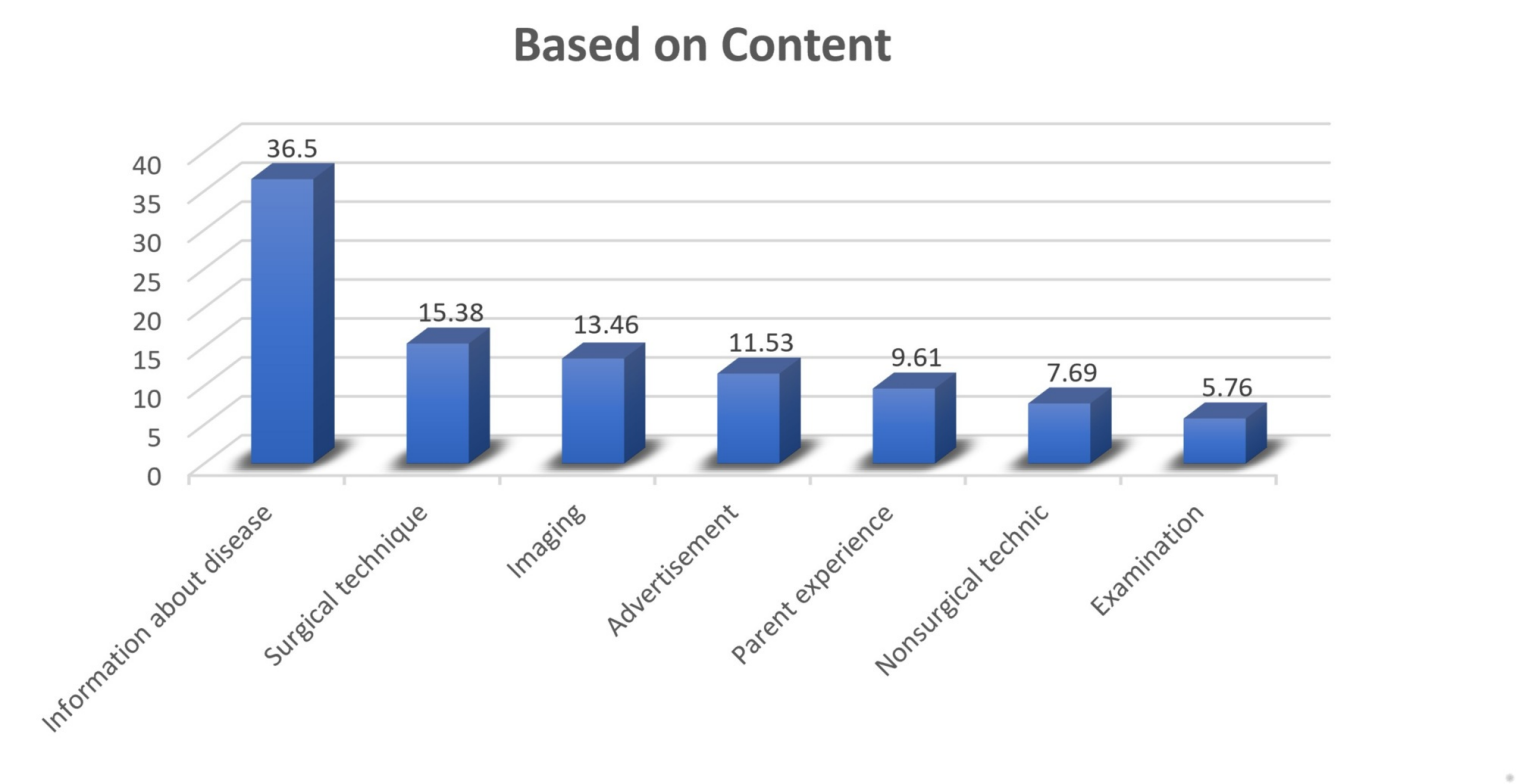
Distribution of the groups based on content

The videos were divided into four groups based on source (academia, physician, parents, and commercial) (Figure 1) and seven groups based on content (information regarding the disease, parent experience, physical examination, non-surgical treatment techniques, surgical treatment techniques, information regarding radiology, and advertisements) (Figure 2).

The Global Quality Score (GQS) and DDH scores (DDHS) were used to evaluate the educational quality of the videos, and the Journal of the American Medical Association Score (JAMAS) was used to evaluate the accuracy of the source of information [7].

The JAMAS uses four criteria to assess the accuracy of the source of the medical information [10]. For each criterion, 1 point was given to each video (Table 1). The quality and educational quality of the videos were assessed using GQS; 1 point was given for poor quality and 5 points were given for excellent quality (Table 2).

**Table 1:** The Journal of American Medical Association benchmark criteria

Criteria	Description	Points
Authorship	Authors and contributors, their affiliations, and relevant credentials should be provided	1 point
Attribution	References and sources for all content should be listed clearly, and all relevant copyright information noted	1 point
Disclosure	Web site “ownership” should be prominently and fully disclosed, as should any sponsorship, advertising, underwriting, commercial funding arrangements or support, or potential conflicts of interest	1 point
Currency	Dates that content was posted and updated should be indicated	1 point

**Table 2:** Global Quality Score for educational value

Score	Description
1	Poor quality; very unlikely to be of any use to patients
2	Poor quality but some information present; of very limited use to patients
3	Suboptimal flow, some information covered but important topics missing; somewhat useful to patients
4	Good quality and flow, most important topics covered; useful to patients
5	Excellent quality and flow; highly useful to patients

As a DDH-specific quality evaluation, we created a new scoring system called DDHS, as did other studies in the literature [7,11]. DDHS is created by taking the literature and current textbooks into consideration. A total of seven topics were created, and for each topic, the videos were given 1 to 3 points if the topic was mentioned in the videos. Zero points were given for no information, 1 point was given for poor information, 2 points were given for moderate information, and 3 points were given if the information was complete (Table 3). Two authors evaluated each video separately, and if the videos received different scores for the scoring systems, they were revaluated and a consensus was reached.

**Table 3:** The scoring system that created for YouTube videos about developmental dysplasia of the hip

Criteria	Description	Points
Spectrum of disease	Dysplasia, subluxation, dislocation…	0 points: no information; 1 point: poor information; 2 points: moderate information; 3 points: complete information
Epidemiology and risk factor	Firstborn, female, breech position, family history, oligohydramnios…	0 points: no information; 1 point: poor information; 2 points: moderate information; 3 points: complete information
Pathophysiology and associated conditions	Development of secondary barriers to reduction, anatomic changes, congenital muscular torticollis, metatarsus adductus, congenital knee dislocation…	0 points: no information; 1 point: poor information; 2 points: moderate information; 3 points: complete information
Physical exam	Barlow Ortolani Galeazzi limitations in hip abduction, Klisic test pelvic obliquity lumbar lordosis, Trendelenburg toe-walking…	0 points: no information; 1 point: poor information; 2 points: moderate information; 3 points: complete information
Imaging	X-ray (Hilgenreiner, Perkins, Shenton line, acetabular index, central edge angle), ultrasound (alpha angle, beta angle, Graf classification), arthrogram, CT, MRI...	0 points: no information; 1 point: poor information; 2 points: moderate information; 3 points: complete information
Treatment	Nonoperative (abduction splint, closed reduction and spina casting), operative treatment (open reduction and spina casting, open reduction and femoral osteotomy, open reduction and pelvic osteotomy)...	0 points: no information; 1 point: poor information; 2 points: moderate information; 3 points: complete information
Complications	Avascular necrosis, recurrence, femoral nerve palsy, limb length discrepancy, coxarthrosis...	0 points: no information; 1 point: poor information; 2 points: moderate information; 3 points: complete information

The interobserver reproducibility was evaluated, and intraclass correlation coefficients (ICCs) were calculated. An ICC value of 0.9 was considered excellent, values between 0.8 and 0.9 were considered good, values between 0.8 and 0.7 were considered moderate, and values below 0.7 were considered poor [12,13].

Statistical analysis

IBM SPSS Statistics Version 22 (IBM Corp., Armonk, NY, USA) was used for statistical analysis. Descriptive statistical methods (mean, standard deviation, median, frequency, percentage, minimum, and maximum) were used to evaluate the study data. The normal distribution of quantitative data was evaluated using the Shapiro-Wilk test and graphical examinations. Student’s t-test was used to compare the two groups with normal distribution. The Mann-Whitney U test was used to compare the two groups of quantitative variables that did not show a normal distribution. The one-way ANOVA (analysis of variance) posthoc multiple comparison Tamhane’s test was used for the comparison of the groups in the case of three or more variables that did not show a normal distribution. The interobserver and intraobserver reproducibility were determined by the ICC. A p-value of <0.05 was considered statistically significant. Pearson’s chi-square test was used to compare qualitative data.

## Results

The mean duration time of the videos was 526 seconds (SD: 813), the average view count of the videos was 34.644, and the mean time since upload was 1,907 days (SD: 1,137). On average, the videos received 10.9 comments, 210.3 likes, and 6.8 dislikes. The mean like ratio and VPI were 92.9 (SD: 19.57) and 25.8 (SD: 53.43), respectively.

On the basis of the source, 49% of the videos were shared by physicians (Figure 1), and on the basis of the content, 36.5% of the videos were regarding information about the disease (Figure 2). The mean JAMAS, GQS, and DDHS of all videos evaluated were 1.37 (SD: 0.7), 2.46 (SD: 1.09), and 4.63 (SD: 5.00), respectively. The DDHS and GQS had a positive correlation (p: 0.001; r: 65.8%). The JAMAS had no correlation with the DDHS or GQS (p > 0.05) (Table 4).

**Table 4:** Results for all the scoring systems based on source and based on content JAMAS, Journal of the American Medical Association Score; GQS, Global Quality Score; DDHS, developmental dysplasia of the hip score; VPI, Video Power Index

	JAMAS, mean+SD (median)	GQS, mean+SD (median)	DDHS, mean+SD (median)	VPI, mean+SD (median)
Video Source				
Academic	1.78±0.87 (2)	3.06±1.25 (3)	7.33±6.80 (4)	33.7±75.1 (1.29)
Physician	1.20±0.40 (1)	2.36±0.86 (2)	3.8±3.20 (3)	23.2±40.2 (7.62)
Parents	1.25±0.5 (1)	0.75±0.5 (1)	0.75±0.5 (1)	18.1±26.2 (7.76)
Commercial	0.80±0.83 (1)	1.60±0.54 (2)	2.20±1.09 (2)	0.05±0.07 (0.05)
Video Content				
Information about the disease	1.26±0.65 (1)	2.95±1.12 (3)	7.32±6.30 (7)	27.8±46.6 (4.97)
Examination	1.33±0.57 (1)	3.67±2.08 (3)	3.67±2.08 (3)	154.7±139.7 (161.5)
Imaging	1±0.57 (1)	5±4.72 (2)	5±4.72 (2)	8.12±5.75 (7.62)
Surgical technic	2±0.75 (2)	3.38±4.03 (2)	3.38±4.03 (2)	13.1±14 (7.06)
Nonsurgical technic	1.5±0.5 (1)	2.5±0.5 (2)	2.5±2.38 (1.5)	0.96±0.47 (1.08)
Advertisement	1±0.89 (1)	1.83±1.32 (2)	1.83±1.32 (2)	0.30±0.44 (0.1)
Parent experience	1.2±0.44 (1)	1.6±0.54 (2)	1.6±1.94 (1)	20.6±23.3 (8.75)
Total	1.37±0.71 (1)	2.46±1.09 (2)	4.63±5.00 (2)	25.7±53.9 (5.88)

In the evaluation based on source, the DDHS was higher in the academic group than in the parent group (p: 0.005) and the commercial group (p: 0.37), and it was higher in the physician group than in the parent group (p: 0.001). There was no significant difference between the other source groups when evaluating the DDHS. The GQS was higher in the academic group than in the parent group (p: 0.02) and the commercial group (p: 0.01). There was no significant difference between the other groups when evaluating the GQS.

In the evaluation based on content, the DDHS was higher in the information about the disease group than in the parent experience group (p: 0.042) and the advertisement group (p: 0.023). There was no significant difference between the other groups when evaluating the DDHS. The GQS was higher in the information about the disease group than in the parent experience group (p: 0.031) and the advertisement group (p: 0.01). There was no significant difference between the other groups when evaluating the GQS.

When evaluating the VPI, like ratio, and view ratio based on source and based on content, a significant difference was observed only in the view ratio. On the basis of the source, the view ratio was higher in the physician group than in the commercial group (p: 0.049).

Interobserver reproducibility was evaluated and ICCs were calculated. In DDHS, the ICC value was 0.78, and in GQS it was 0.62.

## Discussion

YouTube is the most famous online video platform, and its content is growing day by day [14]. YouTube is being used not only for entertainment purposes but also for educational purposes. Patients and the parents of patients are using YouTube to obtain information regarding diseases that they or their children have [15]. DDH is one of the most frequent disorders in newborns and can cause serious complications such as osteoarthritis [16]. Many concerned parents want to learn more about the disease, and they commonly use YouTube to search for information. However, most parents are not capable of evaluating the quality of the medical content of YouTube videos. Poor quality videos may mislead the parents and could impair the relationship between the parents and their physicians. The purpose of this study was to investigate the quality and reliability of YouTube videos regarding DDH.

GQSs are used to evaluate the quality and the educational quality of the videos, and the JAMAS was used to evaluate the accuracy of the source of information [10]. As a DDH-specific quality evaluation, we created a new scoring system called DDHS, as have other studies in the literature [7,11]. We found positive correlations between GQS and DDHS (p: 0.001; r: 65.8%). However, in DDHS, the ICC value was 0.78, and in GQS, it was 0.62. Thus, DDHS provided more objective results than GQS did in the YouTube videos regarding DDH.

There are a number of studies that have investigated the quality of YouTube videos regarding medical information [7,11,17-21]. These studies found that YouTube videos regarding medical information had poor quality. In our study, the mean JAMAS, GQS, and DDHS of the videos were 1.37 (SD: 0.7), 2.46 (SD: 1.09), 4.63 (SD: 5.00), respectively. This result suggests that the videos regarding DDH were of poor quality as well, which is consistent with the literature.

In our study, on the basis of the source, most of the videos (49%) were shared by physicians. In Erdem et al.’s study investigating the quality of YouTube videos regarding kyphosis [7], in Loeb et al.’s [22] study investigating the quality of YouTube videos regarding prostate cancer, and in Ferhatoglu et al.’s [8] study investigating the quality of YouTube videos regarding sleeve gastrectomy, the videos were shared mostly by non-physicians. This difference in the source of the videos could result from the following factors: the diagnosis and the treatment of these three diseases must occur under the supervision of a doctor, there is no natural treatment for the disease, and there is a limited rehabilitation process for the disease.

In our study, in the videos evaluated using VPI scores, there were no significant differences between the groups. By contrast, the literature regarding YouTube videos dealing with medical information has shown that the popularity of the videos decreases when the source of the videos is academic or physicians [4,7,8,11,22]. This inconsistency with the literature shows us that despite demonstrated trends, concerned parents of patients with DDH watched the videos sourced by physicians and academics to obtain more information.

When evaluating the videos based on content, most of the videos were information about the disease (36.5%), and the DDHS and GQS were higher in the information about the disease group than in the parent experience group and the advertisement group. When evaluating the videos based on source, DDHS and GQS were higher in the academic and physician groups than in the parent and commercial groups. This result showed that the videos regarding parent experience and the videos with commercial concerns had poor quality, whereas the videos sourced by an academic or a physician had higher quality, which is again consistent with the literature [7,8,11,22].

There are limitations to this study. First, YouTube is a growing platform. Thus, different results could be obtained if the search was made at a later time. Second, we assessed only the first 52 videos that were returned by YouTube in response to a search for DDH. Although it is a limitation, there is a study in the literature showing that Internet users only consider the first two pages that they obtain when searching for a keyword [23]. Third, we assessed the videos that are returned by YouTube as an answer to the term “developmental dysplasia of the hip” (DDH) only, so as not to divert our study from its purpose. DDH is a relatively a new term for the disease, and parents can also search the disease using the term “developmental hip dislocation”. However, we intended to assess the quality of YouTube videos. Thus, the search for “developmental hip dislocation” should produce similar results as the search for DDH if the videos have medical quality. Fourth, when evaluating the videos with DDHS, the videos regarding a subtitle of DDH could get a minor point because DDHS assesses all of the subtitles of DDH. That is why we evaluated the videos with GQS. Lastly, we assessed only videos that were in the English language.

The videos on YouTube regarding DDH generally had poor quality, which means that the information that parents obtain from YouTube can be misleading, which could be challenging for physicians. Parents and patients have the right to access free and easily accessible information regarding medical situations on the Internet and YouTube. Thus, to maintain the optimal parent-physician or patient-physician relationship, we suggest that international health societies make their own educational videos for parents, patients, and fellow physicians. Videos from proper sources that have high-quality information can be translated into multiple languages to reach more people.

## Conclusions

Our study showed that the videos on YouTube regarding DDH generally had poor quality. Poor quality videos could mislead the parents, and we suggest that international health societies make their own educational videos for parents, patients, and fellow physicians. Correct, easily accessible, and free sources of information are important for maintaining an optimal parent-physician or patient-physician relationship and for achieving the best health outcomes possible.
